# Skin Delivery and *in Vitro* Biological Evaluation of *Trans*-Resveratrol-Loaded Solid Lipid Nanoparticles for Skin Disorder Therapies

**DOI:** 10.3390/molecules21010116

**Published:** 2016-01-20

**Authors:** Roberta B. Rigon, Naiara Fachinetti, Patrícia Severino, Maria H. A. Santana, Marlus Chorilli

**Affiliations:** 1 Faculdade de Ciências Farmacêuticas, UNESP—Universidade Estadual Paulista, Campus Araraquara, Departamento de Fármacos e Medicamentos, Araraquara, SP 14800-850, Brazil; roberta_rigon@yahoo.com.br (R.B.R.); naiara.fachinetti@gmail.com (N.F.); 2 Centro de Ciências Biológicas e da Saúde, Universidade Tiradentes, Aracaju, SE 49010-390, Brazil; pattypharma@gmail.com; 3 Faculdade de Engenharia Química, Universidade Estadual de Campinas, Campinas, SP 13083-970, Brazil; mariahelena.santana@gmail.com

**Keywords:** drug delivery system, skin disorders, permeation, solid lipid nanoparticle, *trans*-resveratrol, tyrosinase

## Abstract

The aim of this study was to evaluate the skin delivery and *in vitro* biological activity of *trans*-resveratrol (RES)-loaded solid lipid nanoparticles (SLNs). The SLNs were composed of stearic acid, poloxamer 407, soy phosphatidylcholine (SPC), an aqueous phase and 0.1% RES. The particle size, polydispersity index (PdI) and zeta potential were analyzed by dynamic light scattering (DLS). The SLNs were analyzed by scanning electron microscopy (SEM-FEG) and differential scanning calorimetry (DSC). *In vitro* RES-SLN skin permeation/retention assays were conducted, and their tyrosinase inhibitory activity was evaluated. An MTT reduction assay was performed on HaCat keratinocytes to determine *in vitro* cytotoxicity. The formulations had average diameter lower than 200 nm, the addition of SPC promoted increases in PdI in the RES-SLNs, but decreases PdI in the RES-free SLNs and the formulations exhibited zeta potentials smaller than −3 mV. The DSC analysis of the SLNs showed no endothermic peak attributable to RES. Microscopic analysis suggests that the materials formed had nanometric size distribution. Up to 45% of the RES permeated through the skin after 24 h. The RES-loaded SLNs were more effective than kojic acid at inhibiting tyrosinase and proved to be non-toxic in HaCat keratinocytes. The results suggest that the investigated RES-loaded SLNs have potential use in skin disorder therapies.

## 1. Introduction

Exposure to solar ultraviolet radiation can induce the formation of free radicals on the surface of human skin, accelerating skin aging, which can be characterized by hyperpigmentation, and may promoting the occurrence of various skin diseases such as melanoma [1]. Studies have demonstrated that skin cancer is caused by a combination of endogenous and exogenous risk factors like solar exposure [2,3]. Human malignant melanoma skin cancer is the most aggressive skin cancer form [4], with the severity of the disease relating to the degree of reactive oxygen species (ROS) in the melanoma cell line [5]. Melanogenesis can affect tumor progression as it promotes an oxidative environment, which is toxic and mutagenic, causing genetic instability and inhibition of immune cells [6,7]. Furthermore, melanin, the final product of melanogenesis, can produce relative hypoxia in the tumor microenvironment due to increased oxygen consumption. Thus, melanogenesis inhibition could be explored as a valid antitumor therapy [8].

**Figure 1:**
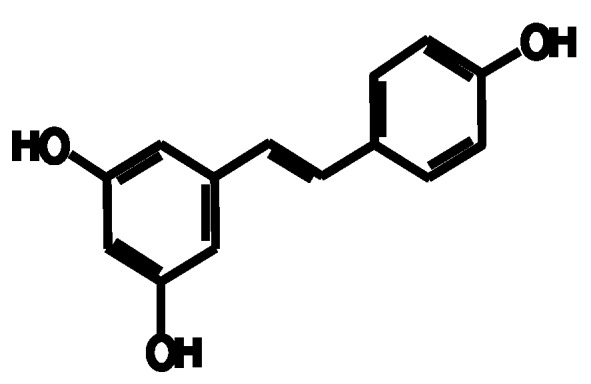
*trans*-Resveratrol structure.

The enzyme tyrosinase is responsible for the first step in melanin production; consequently, it is involved in hyperpigmentation. In pathological states such as melanoma, high levels of tyrosinase in the serum and in tissues promote the overproduction of melanin [9]. Hence, inhibiting tyrosinase activity is a common approach to promote skin whitening, but it could be particularly valuable in the prevention of skin cancers such as melanoma. Resveratrol (*trans*-3,4′,5-trihydroxystilbene) (RES) (Figure 1) is a natural component of grape skins and is notably present in wine [10]. Studies have demonstrated the cancer chemopreventive activity of RES [11]. Kim and coworkers observed that RES is able to reduce tyrosinase activity by 30% to 45% [12]. However, this drug has a low *in vivo* bioavailability when administered orally [1]. Therefore, skin administration may be a convenient method of delivering RES to its site of action.

Solid lipid nanoparticles (SLNs) are a new generation of nanoparticulate drug delivery systems, which are attracting attention as innovative colloidal drug carriers for topical applications [13,14], especially in virtue of their interaction with the *stratum corneum* (SC) and other layers skin. Moreover, SLNS have the ability to protect the drug and control its release [15]. Furthermore, recent studies have shown improved SLN uptake and accumulation in tumor tissue, due to the latter’s physiological characteristics such as abnormalities and dysfunction in the tumor vasculature, which allow SLN to easily permeate the tumor [16]. Moreover, high SLN concentrations are maintained in the tumor for longer periods of time due to low venous return and lymphatic drainage [17,18,19]. The aim of this study was to evaluate the skin delivery and *in vitro* biological activity of *trans*-resveratrol-loaded SLNs, to confirm their potential as skin disorder therapies.

## 2. Results

### 2.1. Development of SLN

The methodology described by Lim and coworkers [20], and Mehnert and Mäder [21] was adapted to produce SLNs by sonication. The formulations developed were composed of 5.0% lipid phase (stearic acid) and 4.7% surfactants (soy lecithin and poloxamer 407). Methylparaben (0.18%) and propylparaben (0.02%) were used as microbiological preservatives. Table 1 describes the compositions of the different SLNs formulations.

**Table 1:** SLN constituents.

	Concentration of Raw Material (%)	
	F1	F2
Stearic Acid (SA)	5.00	5.00
Poloxamer 407 (P407)	3.50	3.50
Soy Lecithin (SL)	-	1.20
Methylparaben	0.18	0.18
Propylparaben	0.02	0.02
Distilled water	q.s *	q.s *

* q.s: indicate quantity sufficient to make 10 mL of formulation. F1.RES and F2.RES have the same lipid formulations as F1 and F2, respectively, but 0.1% of RES was added to the lipid phase.

### 2.2. Hydrodynamic Size of Particles and Zeta Potential Analysis

Table 2 describes the mean hydrodynamic size (Z-Ave), polydispersity index (PdI) and zeta potential (ZP) of the SNLs developed.

**Table 2:** Mean hydrodynamic diameter, polydispersity index and zeta potential of the SLNs (*n* = 3).

	Z-Ave (d.nm)	PdI	ZP (mV)
F1	194.9 ± 1.93 ^a^	0.230 ± 0.01	−1.54 ± 0.31
F2	137.67 ± 1.25 ^b^	0.157 ± 0.02	−2.22 ± 1.12
F1.RES	155.50 ± 0.26 ^c^	0.140 ± 0.02	−2.60 ± 1.27
F2.RES	166.23 ± 0.94 ^d^	0.196 ± 0.02	−2.66 ± 0.33

^a–d^ Different symbols within a column indicate that the differences between these averages are statistically significant (*p* < 0.05).

Statistically significant differences were found between the mean hydrodynamic diameter of the different formulations, both before and after addition of RES.

### 2.3. SLN Morphology

**Figure 2:**
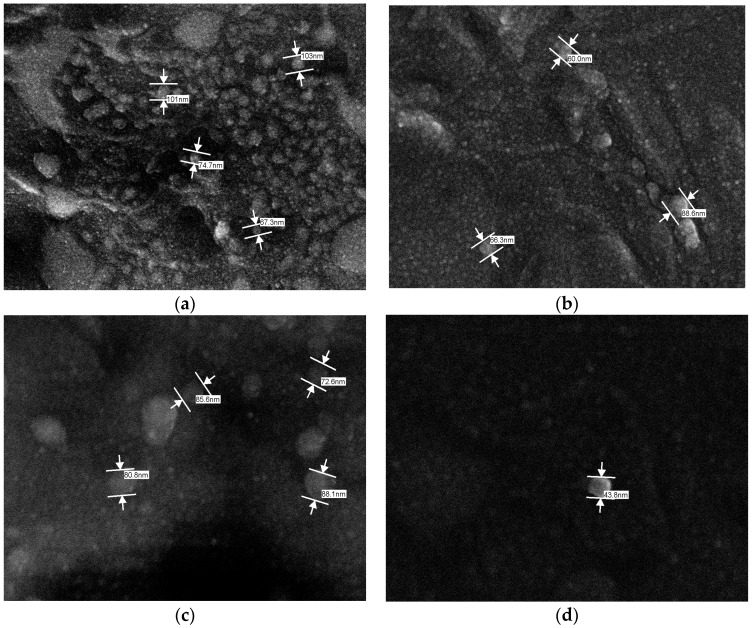
Photomicrograph of formulations: (**a**) F1 (magnified 50,000×); (**b**) F2 (magnified 80,000×) (**c**) F1.RES (magnified 100,000×) and (**d**) F2.RES (magnified 150,000×).

The SLN morphology was obtained using scanning electronic microscopy with a field emission gun (SEM-FEG) as shown in Figure 2.

The photomicrographs demonstrated that SNL solutions presented a low size distribution range, and no crystals were formed. Crystals can be formed when excess compounds that were not entrapped are found in the dispersing medium or because there is a large polydispersity in the particle size.

### 2.4. Characterization by Differential Scanning Calorimetry (DSC)

Thermoanalytical methods are extensively used to analyze the physical proprieties of drugs, such as melting and vaporization temperatures, enthalpies, and glass transitions points, which permits to analyze the compatibility and stability of pharmaceuticals products [22].

**Figure 3:**
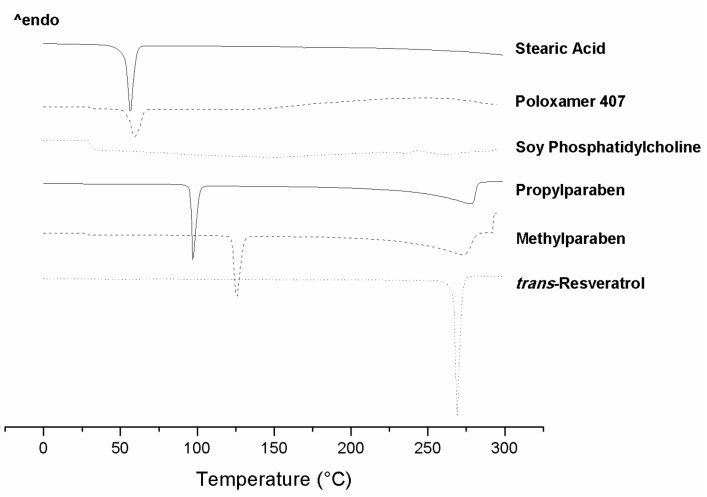
DSC scans of the ingredients used to prepare the SLNs.

**Figure 4:**
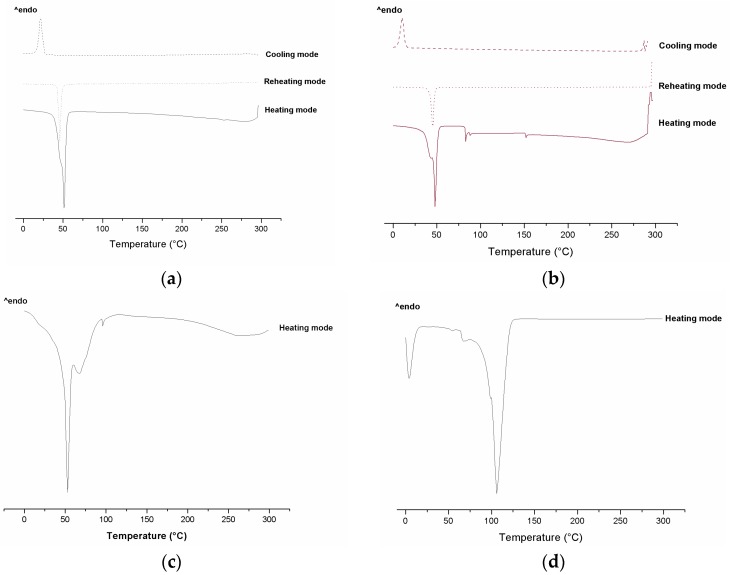
DSC scans of SLNs: (**a**) F1 and (**b**) F1.RES (heating from −50 to 270 °C, subsequent cooling from 270 to −50 °C, and reheating from −50 to 270 °C, at a rate of 10 °C·min^−1^); (**c**) F2 and (**d**) F2.RES (heating from −50 to 270 °C). All formulations were tested at all 3 temperature cycles; however, only the cycles with thermal events are shown. Formulations F2 and F2.RES presented thermal events only in the heating mode.

Figure 3 and Figure 4 present the DSC scans of the individual ingredients used in the SLN preparations and of the SNL formulations with or without addition of RES. The formulations were submitted to calorimetric scans in heating, cooling, and reheating modes. The heating mode verifies the melting point and dehydration of samples and the cooling mode allows to observe the reversibility of the melting process. The reheating mode is performed to observe if the recrystallized formulations present the same melting behaviors as they did originally.

### 2.5. In Vitro Skin Permeation of RES

Determining the ability of different substances to cross the skin is the aim of the skin permeation assay. This test utilizes pig ear skin or human skin obtained from plastic surgery [23,24]. The use of pig ear skin is based on its great anatomical, histological and physiological similarity to human skin, more so than any other common laboratory animal [25]. Table 3 presents the cumulative amount of RES, expressed as mean ± standard deviation (*n* = 6), which permeated through pig skin over 24 h after application of SLN solutions.

**Table 3:** Cumulative amount of RES permeated through pig skin (1.77 cm^2^) after 24 h (temperature maintained at 32 ± 2 °C).

Sample Name	Cumulative Amount (%) after 24 h
F1.RES	45.26 ± 34.88 ^a^
F2.RES	18.61 ± 16.99 ^b^

^a,b^ Different symbols within a column indicate that the differences between these averages are statistically significant (*p* < 0.05).

### 2.6. In Vitro Tyrosinase Inhibition by RES-Loaded SLN

Table 4 presents the tyrosinase inhibitory activity of RES and RES-loaded SLNs.

**Table 4:** Tyrosinase inhibitory activity of RES and RES-loaded SLNs.

	Percentage of Inhibitory Activity (IA%)				
	5 μg·mL^−1^	10 μg·mL^−1^	Equation	R^2^	IA_50_
Kojic Acid	25.06	57.82	y = 6.1282x − 3.102	0.9595	8.66
RES solution	47.04	63.45	y = 0.2314x + 58.208	0.4001	nd *
F1.RES	65.49	89.78	y = 0.9413x + 85.111	0.6163	nd *
F2.RES	55.27	58.93	y = 0.7520x + 57.034	0.8114	nd *

IA_50_ = inhibitory activity at 50%. * nd = not determined.

Kojic acid was adopted as the positive control because it is a potent *in vivo* tyrosinase inhibitor [26].

### 2.7. In Vitro MTT Cytotoxicity Assay of SLN

**Figure 5:**
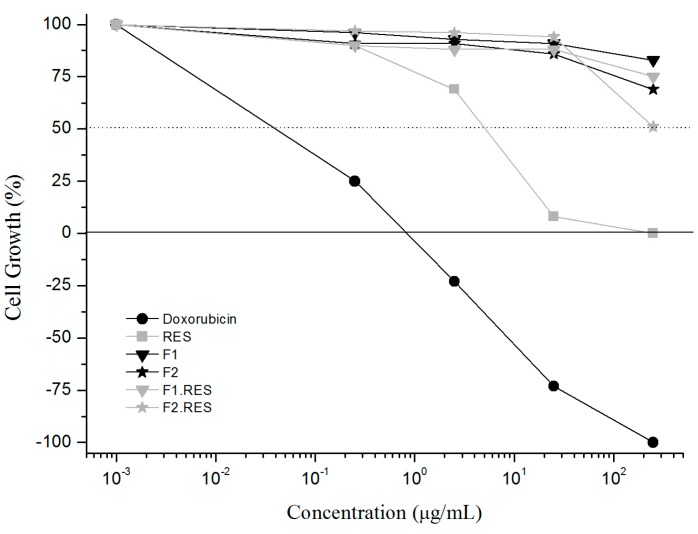
Percentage of cell growth after treatment with positive control (doxorubicin), RES or SLNs.

The cells used in this study were HaCat keratinocytes, a standard cell line for evaluating the *in vitro* cytotoxicity of skin delivery formulations [27,28]. The data are shown as the percentage of cellular growth (Figure 5).

## 3. Discussion

A particle size of 700 nm is ideal for drug permeation. However, there have been many scientific studies demonstrating that particles with more than 700 nm can also permeate the skin [29]. Schaefer and coworkers [30] demonstrated that polymeric particles with diameters in the range of 3 to 10 μm selectively penetrate through follicular ducts, while particles bigger than 10 μm stay under the skin’s surface. Nevertheless, particles with different sizes can also permeate the skin, such as 20 nm polystyrene particles [29].

Particle size is substantial for skin permeation [31]. Shim and co-authors [32] analyzed the *in vitro* skin permeation of minoxidil loaded in 40 nm and 130 nm nanoparticles using pig ear skin. Results demonstrated that smaller nanoparticles facilitated minoxidil skin permeation and recovery in the receptor compartment. This probably occurs because nanoparticles of small size form a homogeneous film on the skin surface that reduces transepidermal water loss (TEWL) and promotes skin permeation of the drug [33]. Furthermore, the presence of lipids in the SLNs can increase their solubility within the stratum corneum, improving drug permeation across the skin barrier [34].

The polydispersity index (PdI) is a parameter used to define the size distribution of particles. Samples with a great range in size distribution present PdI > 0.7 [35]. Dispersions with a PdI equal to 0.4 are considered to have heterogeneous distribution, which may indicate the presence of agglomerates within the sample. Dispersions with PdIs of 0.2 are considered to have minor particle polydispersity [36]. All SLN formulations (Table 2) showed PdIs near to 0.2, demonstrating that these colloids were monodisperse in size.

Schwarz and co-authors [37] developed SLNs composed of Dynasan^®^ 112 (trilaurin), Lipoid^®^ S75 (soy lecithin with 68% of phosphatidylcholine) and poloxamer 188, and they observed that increasing the concentration of SPC decreased the mean particle diameter, but promoted an increase in PdI.

Our results showed similarities with those of Schwarz and co-authors [37]. In relation to the hydrodynamic diameter size the addition of SPC (F2) promoted a reduction in the hydrodynamic diameter size, compared to F1 (Table 2). However, for formulation with RES (F1.RES and F2.RES), the mean diameter increased after addition of SPC (F2.RES). In relation to PdI, the results demonstrated that addition of SPC caused an increase in PdI only in formulation with RES (F2.RES). However, formulations without RES (F1 and F2) presented a decrease in PdI when SPC was added (F2). Akhtar and Pathak have also shown that high SPC concentrations promote lower PdI, probably due to the surfactant activity of this substance [37].

SPC can be composed of different concentrations of mono- and polyunsaturated fatty acids. An increase in the monounsaturated fatty acids ration enhances the particle size. However, long chain polyunsaturated fatty acids contribute to decreasing particle size and to increasing the PdI [38].

The results showed that the formulation presented zeta potential equal to −1.54 ± 0.31 mV and −2.22 ± 1.12 mV for F1 and F2, respectively, and −2.60 ± 1.27 mV and −2.66 ± 0.33 mV for F1.RES and F2.RES, respectively (Table 2). Particles with zeta potentials over +30 mV or under −30 mV are typically considered stable [39]. However, in the preparation of our nanoparticles, steric stabilizer (poloxamer 407) was used for to achieve greater stability, explaining the low zeta potential values that indicate low electrostatic repulsion [40].

According to the photomicrographs, the sonication method produced particles with size distributions on the nanometric scale (Figure 2). The mean particle diameter observed on the photomicrographs was about 100 nm, slightly lower than those found in the dynamic light scattering (DLS) analysis. The DLS evaluates the hydrodynamic size of particles, namely the union between the inorganic core and the solvent layer attached to the particle. When estimating the size by SEM-FEG, this hydration layer is not present; hence, the photomicrographs only project information about the inorganic core. Consequently, the particle size analyzed by DLS is greater than the size estimated by SEM-FEG [41].

The melting peak of SPC was not observed (Figure 3), possibly because SPC presents no steep melting point. Nonetheless, when a melting point is not observed in the DSC scans, it indicates that the substance probably remains in its amorphous form [42]. The DSC scan of poloxamer 407 (Figure 3) revealed an endothermic event at 59 °C and thermal degradation at 150 °C, approximately. Poloxamer 407 can reduce the endothermic events of other substances through their solubilization. As such, these substances are in amorphous form making them invisible on DSC scan of poloxamer 407 containing formulations [43]. Methyl and propylparaben showed unique endothermic events at 129 °C and 97 °C, respectively, which was attributed to melting (Figure 3), these values were consistent with values reported in the literature, which are 124 °C for methylparaben and 97.8 °C for propylparaben [44,45].

The endothermic peak of RES was not observed in the formulations’ thermograms (Figure 4), this may be because RES is stored in an amorphous state within the SNL rather than in a crystalline state, or because high concentrations of lipid impede the detection of the RES melting point by DSC, or because the RES has been dissolved by the stearic acid and poloxamer 407 therefore the crystalline state of RES was undetected [46,47].

Figure 4a shows that F1 and F1.RES presented thermal events in all three analyzed cycles; there is an evident endothermic peak (melting point) in the heating mode, an exothermic peak representing recrystallization in the cooling mode and another melting peak in the reheating mode. This indicates that F1 and F1.RES returned to their initial crystalline state after having been submitted to heating and cooling. The F2 and F2.RES formulations did not present thermal events in the cooling and reheating modes (Figure 4c,d).

The addition of RES to formulation F2 produced an exothermic peak displacement from 50 °C to 106 °C (Figure 4c,d). This could be caused by increased crystal structures or decreased structural defects, promoting the displacement of first endothermic transition to higher temperatures.

The addition of SPC in topical formulations has been associated with an increase in drug skin permeation [48,49,50]. However, the cumulative amount of RES permeated after 24 h was 45.26% ± 34.88% for F1.RES and 18.61% ± 16.99% to F2.RES (Table 3). Therefore, the formulation with SPC (F2.RES) showed lower amounts of permeated RES than the formulation without SPC (F1.RES).

Similar results were observed by Ferderber and co-author [51] that demonstrated a reduction in propranolol skin permeation with increased concentrations of SPC. Bentley and coworkers [52] also demonstrated that the addition of SPC as a skin permeation promoter influences the release profile and cutaneous retention of lipophilic drugs from poloxamer 407 gels. They also conducted a study to evaluate the influence of SPC on skin permeation of triamcinolone acetonide in poloxamer 407 gel. Results demonstrated that an increase in the concentration of SPC promotes a decrease in the amount of drug permeation and increases the drug cutaneous retention [53].

These results could be explained by the fact that phospholipids, such as SPC, promote an increase in drug deposition in the skin, as they are constituents of the SC and can form an extra lipid barrier under the skin surface that decreases the drug flux [54].

RES was not detected in the cutaneous retention assay (dermis, epidermis and *stratum corneum*), signifying that the RES extraction method used on the tape strips and pig skin was not effective. The drug extraction is dependent of the solvent choice. The solvent should not cause drug degradation or solubilize the skin components as this could interfere the analysis [55,56].

The RES solution and formulations F1.RES and F2.RES (5.0 and 10.0 μg·mL^−1^) showed higher percentages of inhibition than kojic acid solution at the same concentrations. At 5.0 μg·mL^−1^, the RES solution, F1.RES and F2.RES inhibited tyrosinase 1.87, 2.61 and 2.20 times more than kojic acid, respectively; whilst, at 10.0 μg·mL^−1^, the RES and F1.RES solutions inhibited tyrosinase 1.10 and 1.55 times more than kojic acid, respectively, while the F2.RES solution had a similar effect to kojic acid (Table 4).

The results demonstrated that formulations F1.RES and F2.RES showed greater tyrosinase inhibition activity than RES solution at both concentrations (5.0 and 10.0 μg·mL^−1^), demonstrating that incorporating RES into SLNs potentiates the inhibitory activity of RES. Furthermore, F1.RES (5.0 and 10.0 μg·mL^−1^) was the most effective formulation.

The IA_50_ values of RES solutions, F1.RES and F2.RES, cannot be calculated because there is no linear relationship between the RES solution, or the SLN solutions, and percentage of inhibition of mushroom tyrosinase activity (R^2^ < 0.90), as shown in Table 4. However, the IA_50_ value of kojic acid was calculated to be 8.66 μg·mL^−1^.

The cytotoxicity of the different SLN formulations was determined by calculating the ratio of viable cells in the SLN-treated wells to those in the untreated control wells (negative control). Results are presented as the percentage of viable cells. Thus, high percentages of cellular viability indicate low toxicity (10%, high toxicity; 11% to 40%, moderate toxicity; 40% to 70%, low toxicity; ≥70% no toxicity) [57].

The RES and doxorubicin (DOX) solutions decreased viability of HaCat cells by 50% at concentrations of 4.4 and 0.6 μg·mL^−1^, respectively (Figure 5). Figure 5 shows that none of the SLN formulations were cytotoxic up to concentrations of 25.00 μg·mL^−1^, maintaining 85% of cellular viability. Although at F2 and F2.RES concentrations of 250 μg·mL^−1^, the cell viability was reduced to 69% and 51%, respectively. F1 and F1.RES presented no cytotoxic effect at high concentrations (250 μg·mL^−1^), as cell viability remained at 83% and 75%, respectively. During our tests, it was observed that the reduction in cell viability with F2 and F2.RES treatment occurred due to the presence of SPC in these formulations, which increased the viscosity of the media, promoting insufficient oxygen supply and hypoxia. However, no concentration of SLN promoted high toxicity, only low toxicity was observed with F2 and F2.RES.

## 4. Materials and Methods

### 4.1. Materials

*trans*-Resveratrol 99% (Sigma-Aldrich, Steinheim am Albuch, Germany), batch number #030M5216V, was used as standard chemical substance. Stearic acid (Via Farma, Joinville, Brazil), soy phosphatidylcholine (Epikuron^®^ 200, Lucas Meyer, Champlan, France), poloxamer 407 (Pluronic^®^ F127, Sigma-Aldrich, St. Louis, MO, USA), methylparaben (Nipagin^®^ M, PharmaSpecial, Santana de Parnaíba, Brazil), propylparaben (Nipasol^®^ M) and *trans*-resveratrol 99% (Resveratrol extract 100%, Galena^®^ Química e Farmacêutica, Campinas, Brazil) were used to prepare SLNs. Polysorbate 80 (Sigma-Aldrich), CLAE methanol (J.T. Baker, Center Valley, PA, USA), transparent scotch tape 750 (Scotch^®^ 750, 3 M Brazil, Sumaré, Brazil), L-tyrosine (Sigma-Aldrich) and mushroom tyrosinase (Sigma-Aldrich) were used to conducted the *in vitro* skin permeation and tyrosinase inhibitory activity assay.

### 4.2. Methods

#### 4.2.1. Development of SLN

For all formulations, the lipid phase (solid lipid + soy lecithin + *trans*-resveratrol) was heated to approximately 5–10 °C above its melting point (~70 °C), before being added to the aqueous solution (P407 solution + methylparaben and propylparaben) of the same temperature. The formulations were stirred for 1 min using a magnetic stirrer. Then, the SLN mixture was sonicated using an ultrasonic processor (Branson Sonifier 250, Branson Ultrasonics Corporation, Danbury, CT, USA) for 20 min (amplitude 47%, 500 W power, 1/2 inch probe). During sonication, samples were maintained in a cooling bath. Since the sonication process can eliminate titanium, samples were centrifuged at 5000 rpm for 10 min [58].

#### 4.2.2. Hydrodynamic Size of Particle and Zeta Potential Analysis

The size of the nanoparticles was determined by dynamic light scattering (DLS, Zetasizer Nano NS, Malvern Instruments, Malvern, UK). Prior to evaluating the mean diameter of the particles and the polydispersity index, the samples were diluted in ultra-purified water to attenuate their opalescence. The zeta potential of the lipid nanoparticles was also measured in purified water, adjusting the conductivity (50 μS·cm^−1^) with a solution of potassium chloride (0.1%). The zeta potential was determined from the electrophoretic mobility using the Helmholtz-Smoluchowski equation. The processing was performed using the software included in the system.

#### 4.2.3. SLNs Morphology

The SLNs morphology was realized by scanning electronic microscopy with field emission gun (SEM-FEG; JSM-7500F, JEOL, Akishima, Tokyo, Japan). A drop of each sample was applied to a silicon substrate and dried in a vacuum desiccator for 12 h. Each sample was coated with carbon (Sputter Coater SCD 050) and analyzed at 10 and 20 kV.

#### 4.2.4. Characterization by Differential Scanning Calorimetry (DSC)

DSC analysis was performed using a Q10 DSC calorimeter (TA Instruments, New Castle, DE, USA) equipped with a liquid nitrogen cooling system (LNCS). The individual components of the SLNs and the SLNs, hermetically sealed in aluminum pans, were submitted to calorimetric scans in heating, cooling and reheating modes. The scans were performed at a temperature range of −50 to 270 °C. The scan rate in the heating and reheating modes was 10 °C·min^−1^. A nitrogen atmosphere with a flow rate of 50 mL·min^−1^ was utilized.

#### 4.2.5. High-Performance Liquid Chromatographic (HPLC) Analysis of RES

RES quantification was performed by reversed-phase HPLC with photodiode array (PDA) detection (Waters^®^ 2695 Alliance quaternary pump, Waters^®^ 2996 photodiode array detector, Waters, Milford, MA, USA) and a RP-C18 Luna column (250 mm × 4.6 mm I.D. 5 µm; Phenomenex, Torrance, CA, USA) that was maintained at 24 ± 1 °C. The mobile phase gradient was the following: water: acetonitrile (75:25, *v*/*v*) from 0 to 3.5 min; water–methanol–acetonitrile (32.5:30.0:37.5, *v*/*v*) from 3.6 to 5.8 min and water–acetonitrile (75:25, *v*/*v*) from 5.9 to 10 min. The flow rate was set to 1.0 mL·min^−1^. RES was detected at the wavelength of 306.6 nm. The mean recovery of RES was 96.84% ± 0.32% and the method was robust for change in the flow rate of mobile phase and column temperature. The intra- and inter-assay coefficients of variation were less than 5%. The drug-free SLN formulation did not exhibit any peak at RES retention time for all formulation studied.

#### 4.2.6. *In Vitro* Skin Permeation of RES

The *in vitro* RES skin permeation was measured using a Franz diffusion assembly. Pig ear skin was mounted between the donor and acceptor compartments, wherein the SC was kept in contact with the formulation and the dermis in contact with the receptor solution [59]. The donor medium consisted of 0.3 mL of SNL solution. The receptor medium (7 mL) was an aqueous solution of 2.0% polysorbate 80, which maintained proper skin conditions. The stirring rate and temperature were kept at 300 rpm and 32 ± 2 °C. Samples (1.5 mL) were collected from the receptor compartment at the appropriate intervals (1, 2, 4, 6, 8, 12, 16, 20, and 24 h) and immediately replaced with fresh receptor medium. The permeated amount of RES was determined by HPLC.

After removing the residual formulation on the skin surface, the SC was removed by 15 successive tape strippings using Scotch tape strips (750 Scotch^®^ Transparent Tapes, 3M Brazil). The first strip was discarded and the 15 following strips were collected in tubes containing 5.0 mL of methanol, vortexed for 2 min, and then placed under ultrasound for 30 min. The remaining pig skin was cut into small pieces and placed in a tube containing 5 mL of methanol; these samples were then subjected to the same procedure in order to evaluate SNL retention within the epidermis and dermis [60]. The amount of RES present in the SC and in the remaining pig skin was determined by HPLC.

#### 4.2.7. *In Vitro* Tyrosinase Inhibition by RES-Loaded SLNs

The enzymatic reaction described by Kobayashi and coworkers [61] was used to analyze the *in vitro* tyrosinase inhibition [62]. Phosphate buffer (70 μL, pH 6.8), tyrosine solution (500 μL, 0.3 mg·mL^−1^ in water), tyrosinase solution (5.0 μL, 480 U·mL^−1^ in phosphate buffer), as well as water (blank, 60 μL), SLNs without RES (control), SLNs with RES (F1.RES and F2.RES) or RES solution (0.312; 0.625; 1.25; 2.5; 3.0; 4.0; 5.0; and 10.0 μg·mL^−1^), were mixed in a micro-tube. The solution was stirred at 30 ± 1 °C for 60 min. The UV absorbance of the solutions were measured at 490 nm. Each sample was analyzed eight times. The results obtained were compared with 5.0 and 10.0 μg·mL^−1^ kojic acid. The percentage of tyrosinase inhibition was calculated using Equation (1):

[(A − T0) − (B − T0)/A] × 100(1)

where A: control absorbance; B: sample absorbance; T0: absorbance at time zero. The IA_50_ value was calculated by varying the concentration of the test substance.

#### 4.2.8. *In Vitro* SLN Cytotoxicity Assay Using 3-(4,5-Dimethylthiazol-2-yl)-2,5-diphenyltetrazolium Bromide (MTT) in HaCat Keratinocytes

*In vitro* cytotoxicity assays for F1.RES and F2.RES were performed using HaCat keratinocytes as a skin cell model. Cells were cultured in DMEM medium with 10% (*v*/*v*) fetal bovine serum (FBS) and penicillin/streptomycin (100 IU/mL/100 μg·mL^−1^). Cells were seeded in 96-well plates at a density of 10^3^ cells/well and incubated with different concentrations of RES or SLN (0.001, 0.25, 2.5, 25.0 and 250.0 μg·mL^−1^) at 37 °C and 5.0% of CO_2_ for 24 h, or with the same concentrations of doxorubicin as a positive control. After removal of the treatment media, the cells were washed with PBS and cell viability was assessed using the MTT reduction assay [60,63]. The MTT test consists of measuring the amount of 3-(4,5-dimethylthiazol-2-yl)-2,5-diphenyltetrazolium bromide (MTT) reduced by active mitochondria. Viable cells are able to reduce MTT to insoluble formazan, which is purple in color when dissolved in an appropriate solvent. The coloration intensity is proportional to the number of viable cells and can be measured by spectrophotometry at 570 nm [64].

#### 4.2.9. Statistical Analysis

The analysis of variance (ANOVA) followed by a Tukey test were carried out on parametric data using Origin 12.5 software (OriginPro 8 SRO, OriginLab Corporation, Northampton, MA, USA). Statistical relevance was assumed at *p* < 0.05.

## 5. Conclusions

The obtained results suggest that the SLN formulations developed could be used for RES administration, improving their efficacy in skin disorder therapies, as both formulations (F1.RES and F2.RES) showed greater tyrosinase inhibitory activity than RES solution, suggesting that incorporating RES in SLNs potentiates the inhibitory activity of RES. The RES containing SLNs also demonstrated a capacity to inhibit tyrosinase activity, greater than or equal to that of kojic acid. All the formulations were demonstrated to be non-toxic in HaCat keratinocyte; as such, they have potential use in therapies against skin disorders like aging and hyperpigmentation.

## Abbreviations

The following abbreviations are used in this manuscript:

RES: *trans*-resveratrol
SLN: solid lipid nanoparticle
SA: stearic acid
SPC: soy phosphatidylserine
DLS: dynamic light scattering
SEM-FEG: scanning electron microscopy with field emission gun
DSC: differential scanning calorimetry
HPLC-PAD: high-performance liquid chromatographic with photodiode array
SC: *stratum corneum*
Z-Ave: mean hydrodynamic size
PdI: polydispersity index
TEWL: transdermal water loss
IA_50_: 50% inhibitory activity
DOX: doxorubicin
DMEM: Dulbecco’s Modified Eagle's Medium
FBS: fetal bovine serum
MTT: 3-(4,5-dimethylthiazol-2-yl)-2,5-diphenyl tetrazolium bromide
ANOVA: analysis of variance
